# Lexical retrieval in fluent and nonfluent aphasia: a network analysis of verbal fluency data

**DOI:** 10.3389/fnhum.2025.1710907

**Published:** 2025-12-05

**Authors:** Catherine T. Pham, Nichol Castro, Jiyeon Lee

**Affiliations:** 1 Department of Speech, Language, and Hearing Sciences, Purdue University, West Lafayette, IN, United States; 2 Department of Communicative Disorders and Sciences, University at Buffalo, Buffalo, NY, United States

**Keywords:** aphasia, lexical retrieval, verbal fluency, semantic network, graph theory, language

## Abstract

Lexical retrieval is commonly impaired in many persons with aphasia (PWA). Verbal fluency tasks are often used to assess lexical retrieval ability. However, common methods of analyzing verbal fluency data (e.g., total number of appropriate responses, clustering and switching) fail to adequately capture the underlying organization of the mental lexicon. To better understand the nature of lexical-semantic organization in aphasia, this study applied a semantic network approach to verbal fluency data obtained from 120 healthy controls and 127 PWA (64 fluent and 63 nonfluent). Participants named as many animal category members as they could in 1 min, and their responses were converted into semantic networks. Global network metrics were computed for each group, including average shortest path length, clustering coefficient, and modularity. Compared to the healthy control network, the PWA network was less integrated and more fragmented, reflected by longer average shortest path lengths, reduced clustering, and higher modularity. These disruptions were especially evident in the nonfluent PWA network compared to the fluent PWA network. Complementary spreading activation and percolation analyses demonstrated that PWA networks were both less efficient and less resilient to disruption. Our results demonstrate that network-based analyses of verbal fluency provide a sensitive measure of lexical-semantic organization in aphasia, revealing structural disruptions that are not fully captured by traditional analyses. More broadly, this approach highlights how network science can advance theories of lexical-semantic organization and inform the development of individualized clinical assessments and treatment strategies.

## 1 Introduction

Aphasia is a language disorder that commonly follows stroke or other neurological injuries. Individuals with aphasia often struggle to produce and/or comprehend language despite intact conceptual knowledge, leading to significant communication challenges. Impaired lexical retrieval is a pervasive deficit observed in persons with aphasia (PWA; Bose et al., 2017) and other neurological populations (e.g., Alzheimer’s disease; Wright et al., 2023). Traditional neuropsychological assessments and linguistic analyses have provided valuable insights into these deficits, yet they often fail to capture the complex, interconnected nature of lexical access. Recent advancements in computational modeling and network science offer new opportunities to analyze lexical retrieval through the lens of semantic network structure. By examining the semantic organization and connectivity of words produced in verbal fluency tasks, researchers can gain a deeper understanding of how lexical access is disrupted in aphasia.

### 1.1 Models of lexical retrieval in aphasia

Most models of lexical retrieval posit at least two stages: (1) lemma (word) retrieval and (2) phonological retrieval (for a review, see Levelt, 1999). These models often employ a spreading activation process (Anderson, 1983; Collins and Loftus, 1975), which emphasizes the interconnected nature of lexical representations. For example, in interactive models of lexical retrieval (Dell et al., 1997; Foygel and Dell, 2000; Rapp and Goldrick, 2000), lexical representations are organized as a network of semantic, lexical, and phonological nodes. Activation of one node spreads to other nodes through weighted connections, with the opportunity for bidirectional and interactive spread of activation across levels of the network. To date, the study of lexical retrieval impairment in aphasia has been heavily influenced by interactive models (e.g., Dell et al., 1997, 2004; Foygel and Dell, 2000; Martin and Dell, 2019; Rapp and Goldrick, 2000).

Lexical retrieval failures can stem from storage-related deficits (i.e., loss of lexical representations) and/or access-related deficits (i.e., difficulty retrieving intact lexical representations; Mirman and Britt, 2014; Rapp and Caramazza, 1993; Warrington and Shallice, 1984). In acquired aphasia (e.g., post-stroke aphasia), the prevailing hypothesis is that lexical retrieval failures reflect access-related deficits (Mirman and Britt, 2014). That is, the lexical representations of words are present in the mental lexicon, but aphasia disrupts the ability to successfully retrieve these words. Access-related deficits include difficulties in the maintenance and/or transmission of spreading activation (Foygel and Dell, 2000; Martin and Dell, 2019), resulting in lexical retrieval failures or speech errors.

To study lexical retrieval in aphasia, commonly used clinical and research tools often include confrontation (picture) naming tasks, where individuals retrieve the correct word for a pictured object or action. These tasks provided a controlled and well-established method for assessing lexical access and are generally sensitive to word-finding difficulties. However, because they typically focus on isolated word production, they are limited in their ability to capture the dynamic, relational structure of the mental lexicon. Given that interactive models emphasize the spread of activation across interconnected representations, examining lexical retrieval abilities utilizing tasks that can capture the organization and spread of activation of the lexical-semantic system can provide further insight into how these systems break down in aphasia.

### 1.2 Verbal fluency as a measure of lexical retrieval

Verbal fluency tasks offer a complementary approach to confrontation naming, one that is slightly less constrained and more reflective of how words are connected and activated in the mental lexicon. During a verbal fluency task, participants produce as many unique exemplars as possible within a specified time limit (e.g., produce as many animals as possible in 1 min). Unlike picture naming or word repetition tasks, which isolate retrieval at the level of individual word selection, verbal fluency requires dynamic word generation under broader category (e.g., animals) or phonemic (e.g., words starting with a certain letter) prompts, providing a window into the temporal and relational links between words. In other words, this approach allows us to examine how access of one word leads to the next and how participants move through their semantic space. This approach also captures retrieval difficulties not only in terms of activation strength but also in the ability to sustain and navigate lexical-semantic networks over time. By analyzing how PWA generate words in a sequential manner, verbal fluency tasks can potentially reveal impairments in network structure (e.g., unexpected connections between words) and search strategies (e.g., how activation spreads from one subcategory, like pets, to another subcategory, like zoo animals), offering critical insights into the mechanisms underlying lexical retrieval failures in aphasia. Deficits in verbal fluency performance have long been observed in individuals with aphasia (Adams et al., 1989; Arroyo-Anlló et al., 2012; Baldo et al., 2010; Basso et al., 1997; Bose et al., 2017, 2022; Faroqi-Shah and Milman, 2018; Niemkiewicz et al., 2022; Roberts and Dorze, 1994), as well as in other neurological populations (e.g., Alzheimer’s disease; Henry et al., 2004; Wright et al., 2023), reflecting impairments in lexical retrieval, semantic network organization, and cognitive flexibility.

Verbal fluency tasks are widely used to assess lexical retrieval ability. They are commonly incorporated into standardized language and/or cognitive assessments (e.g., Western Aphasia Battery-Revised: Kertesz, 2006; Cognitive Linguistic Quick Test-Plus: Helm-Estabrooks, 2017) and have also been used to evaluate treatment efficacy (Beeson et al., 2011; Henry et al., 2008; Kiran et al., 2011), making them a critical tool in both research and clinical contexts. Traditional scoring of verbal fluency has typically focused on broad metrics, such as the total number of appropriate items, which offers a practical and scalable way to index overall lexical access. However, this broad metric fails to capture the complexity of lexical retrieval or the structural organization of the mental lexicon. Furthermore, traditional scoring often lacks sensitivity to distinguish between different patient populations (Henderson et al., 2023; Scheffel et al., 2021). Most importantly, only quantifying the total number of appropriate responses does not fully capture one of the most compelling features of verbal fluency performance: the way words in a sequence are connected to each other in the mental lexicon.

Verbal fluency not only reveals *what* words are retrieved but also *how they are linked*. There are finer-grained analytic techniques, including analysis of the time-course of productions (Bose et al., 2022; Friesen et al., 2015; Luo et al., 2010; McDowd et al., 2011; Sandoval et al., 2010) and clustering and switching behaviors (Bose et al., 2017, 2022; Troyer, 2000; Troyer et al., 1997, 1998), that move closer to capturing the relational aspects of lexical retrieval (i.e., how one word cues or constrains the next). While valuable, these analyses can be time and labor intensive, often requiring manual annotation and scoring. Furthermore, clusters, in particular, are quite subjective in delineation and can be defined based on a number of dimensions. For example, Troyer et al. (1997) provide a detailed protocol for determining clusters and switches for the category *animals* based on subcategories such as taxonomy or living environment. Ledoux et al. (2014) expand upon Troyer et al.'s (1997) protocol to capture additional strategies used during lexical retrieval, like associative and phonemic relationships between successive exemplars during a semantic fluency task. Thus, in addition to resource demands, differences in scoring methods leads to subjectivity and reduced comparability across studies (Abwender et al., 2001; Olabarrieta-Landa et al., 2017; Ross, 2003; Ross et al., 2007), limiting their use in clinical and research settings. These limitations of commonly utilized analysis methods of verbal fluency data highlight the need for complementary analytic approaches that can preserve the rich, connection-based information embedded in verbal fluency responses while offering greater objectivity and scalability.

#### 1.2.1 The value of a network science approach

Network science provides a more objective method for analyzing semantic fluency data compared to traditional analyses and offers a powerful set of computational tools to analyze verbal fluency data as interconnected networks, providing insights into how words are organized and accessed (Kenett et al., 2013; Lebkuecher et al., 2024; Niemkiewicz et al., 2022; Zemla, 2022). One key advantage of semantic network analysis is its ability to represent the underlying structure of lexical retrieval without relying on predefined categories but instead focuses on the emergent structure of the verbal fluency output based on the sequence of words produced. This reduces subjectivity and provides a clearer picture of semantic organization and access across individuals. Semantic networks can be constructed from words generated using a variety of tasks, including semantic fluency tasks (e.g., Cosgrove et al., 2021; Kenett et al., 2013; Lebkuecher et al., 2024; Siew and Guru, 2023; Zemla, 2022). In these networks, nodes represent individual words, while edges define relationships between them, typically based on semantic similarity or co-occurrence patterns (Siew et al., 2019). By applying network science principles, researchers can quantify lexical organization and retrieval efficiency by examining distinct network properties (Barabási, 2013).

Semantic networks can be examined at multiple levels to allow for robust and quantifiable metrics of lexical organization (see Siew et al., 2019). For instance, semantic networks offer access to both *local properties* such node degree (the number of connections a given word) and local clustering coefficient (local CC; the likelihood that neighbors of an individual node will also be neighbors with each other) as well as *global properties* like average shortest path length (ASPL; the average length of the shortest path between any pairs of nodes), global clustering coefficient (global CC; the average likelihood that neighbors of a node will also be neighbors with each other), and modularity (Q; how well communities, or subgroups of nodes, are separable from each other in a network). By leveraging graph theory, semantic network approaches complement traditional approaches by providing a systems-level perspective on how lexical items are structured and navigated during word generation, allowing researchers to capture the dynamic, multidimensional nature of lexical retrieval.

In recent years, there has been emerging interest in utilizing network science to analyze verbal fluency data in clinical populations. It has been found that the semantic networks of individuals with Alzheimer’s disease tend to be smaller in size (i.e., fewer nodes) with a denser, more random structure than healthy controls, suggesting more spurious associations between words (Arias-Trejo et al., 2021; Bertola et al., 2014; Lerner et al., 2009; Zemla and Austerweil, 2019). Additionally, network measures also correlated with performance on the Mini-Mental State Examination (MMSE; Folstein et al., 1975), such that individuals with Alzheimer’s disease who scored worse on the MMSE produced fewer number of nodes and more dense networks (Bertola et al., 2014; Zemla and Austerweil, 2019). A few studies have also utilized a network science approach to investigate word-finding difficulties in patients with multiple sclerosis (MS) using semantic fluency data. Abad et al. (2015) found that neurotypical adults and those with MS displayed small-world properties and comparable community structures in their estimated networks. However, the MS group showed a sparser network with fewer connections between nodes and lower overall interconnectivity. More recent work by Lebkuecher et al. (2024) also reported reduced interconnectivity in networks of MS patients relative to controls, evidenced by lower local and global clustering coefficients, longer average shortest path length, and higher modularity compared to healthy controls. They also found that the MS network was less resilient and less flexible compared to the healthy control network. While previous studies have applied network science to examine lexical retrieval processes in aphasia, they have primarily focused on modeling production errors in naming tasks (Baker et al., 2023; Castro et al., 2020; Castro and Stella, 2019; Vitevitch and Castro, 2015). To our knowledge, no studies have utilized this methodology to analyze semantic fluency data.

### 1.3 The current study

A semantic network approach to analyzing verbal fluency data enhances objectivity, sensitivity, and interpretability by modeling the structure of lexical retrieval rather than simply counting words or identifying broad clusters. To better understand the nature of lexical-semantic organization in aphasia, this study applies a semantic network approach to verbal fluency data obtained from a large group of 120 healthy controls and 127 persons with aphasia, representing a considerably larger sample size compared to previous research examining verbal fluency data in aphasia. We aim to examine (a) differences in lexical-semantic network structure between healthy controls and adults with post-stroke aphasia and (b) differences in the lexical-semantic networks of different clinical aphasia profiles, specifically fluent vs. nonfluent aphasias.

The focus on fluent and nonfluent PWA is driven by findings that individuals with nonfluent aphasia produce fewer responses during verbal fluency tasks (Bose et al., 2017), while individuals with fluent aphasia are often able to produce words more readily, resulting in a greater number of responses, albeit still below neurotypical levels (Baldo et al., 2010). Further, traditional clustering and switching analyses have mixed results between fluent and nonfluent aphasia. Baldo et al. (2010) reported that a patient with Wernicke’s aphasia, which is traditionally categorized as a type of fluent aphasia, produced abnormally small clusters of animal exemplars with more erratic switching, while a patient with Broca’s aphasia, which traditionally categorized as a type of nonfluent aphasia, produced an average cluster size within normal range, despite generating fewer words overall. However, Bose et al. (2017) found no significant differences in cluster size or number of switches when comparing fluent (n = 11) and nonfluent (n = 17) PWA, suggesting that clustering and switching analyses may lack the sensitivity to capture subgroup differences in lexical-semantic organization. These mixed findings highlight the need for complementary approaches, such as semantic network analysis, that model the structure of lexical relationships more directly and quantitatively.

To our knowledge, this is the first study to apply semantic network analysis to verbal fluency data in aphasia. We constructed group-level semantic networks and compared global network metrics (average shortest path length, clustering coefficient, and modulatory) across groups. We note that our method of network construction captures group-level co-occurrence structure, not temporal order of individual responses. Consequently, two items contribute equally to an edge regardless of whether they occurred consecutively or many positions apart. Network analyses that focus on temporal dynamics address a complementary question that have shown important effects of sequence structure (Bertola et al., 2014; Zemla and Austerweil, 2018, 2019), but in the current study, we focus on semantic structure over temporal dynamics. We predicted that PWA networks would exhibit signs of degraded connectivity relative to healthy controls, reflected by longer path lengths (indicating less efficient connections between concepts), lower clustering (indicating weaker local semantic groupings) and higher modularity (indicating more fragmented or segregated substructures). We further predicted that network disruption would be most severe in the nonfluent group. Specifically, nonfluent PWA networks were expected to show even longer ASPL and higher modularity than fluent PWA networks, consistent with more isolated word clusters and fewer cross-cluster links. Fluent PWA networks were also expected to show relatively higher CC and short ASPL than nonfluent PWA networks, aligning with their greater fluency and switching capacity. In sum, we anticipated a gradation in semantic network integration, from intact in controls, to moderately disrupted in fluent PWA, to more severely impaired in nonfluent PWA.

To further disentangle whether lexical retrieval difficulties stem from degradation of semantic structure or from impaired access to otherwise intact representations, we also implemented spreading activation and percolation analyses. Spreading activation simulations estimate how efficiently activation propagates from initially activated nodes to neighboring nodes in the network (Siew, 2019), a core feature of interactive models of lexical retrieval (Dell et al., 1997; Foygel and Dell, 2000; Rapp and Goldrick, 2000). Reduced/slower spreading activation reflects impaired access. Percolation analysis quantifies how easily a network fragments when nodes are systematically removed (Cosgrove et al., 2021). Network structures that break apart more rapidly under percolation reflect structural degradation, a loss of connectivity or organization in the semantic system itself. While spreading activation models the efficiency of access to existing representations, percolation analysis reveals vulnerability of the underlying structure. Together, these methods allow us to move beyond surface-level performance metrics and examine both how and why lexical retrieval may break down in aphasia.

## 2 Materials and methods

### 2.1 Participants

Participants consisted of 120 cognitively healthy controls (HC; 82 females, 38 males) and 127 persons with post-stroke aphasia (PWA; 55 females, 72 males). See Table 1 for a summary of HC and PWA demographic information. All PWA were at least 6 months post onset of stroke at the time of testing, except for 3 PWA who were at 3–4 months post onset (*M_post-stroke_* = 60.28; *SD _post-stroke_* = 60.28; Min *_post-stroke_* = 3; Max *_post-stroke_* = 295). PWA were categorized as fluent (N = 64; 30 females, 34 males; 54 Anomic, 6 Conduction, 4 Wernicke’s) or nonfluent aphasias (N = 63; 25 females, 38 males; 46 Broca’s, 1 Global, 16 Transcortical Motor) based on WAB-R classifications. See Table 2 for a summary of Fluent and Nonfluent demographic information. We conducted independent samples *t*-tests to examine whether HC and PWA differed in age and education. HC (*M_age_* = 65.57, *SD_age_* = 9.88) were significantly older than PWA (*M_age_* = 59.50, *SD_age_* = 13.17; *t*(233.19) = 4.11, *p* < 0.0001). HC also had significantly more years of education on average (*M_edu_* = 16.49, *SD_edu_* = 2.34) than PWA (*M_edu_* = 15.70, *SD_edu_* = 2.32; *t*(237.87) = 2.62, *p* < 0.01). All participants were adult monolingual native English speakers with no reported history of acquired neurological (e.g., dementia) conditions, other than left hemisphere stroke for PWA. Additionally, all HC scored within normal limits on the composite severity ratings of the CLQT+ (Helm-Estabrooks, 2017), which encompasses subdomains for language, executive function, memory, attention, and visuo-spatial skills. All participants provided informed written consent prior to study participation and were compensated for their time. All procedures were institutional review board approved.

**Table 1:** Summary of HC and PWA demographics.

	HC (*N* = 120)				PWA (*N* = 127)			
	*M*	*SD*	Min	Max	*M*	*SD*	Min	Max
Age	65.57	9.88	38.45	84.49	59.50	13.17	24.42	91.92
Education (Years)	16.49	2.34	12	23	15.70	2.32	11	22

**Table 2:** Summary of PWA demographics and WAB-R SCORES.

	Fluent PWA (*N* = 64)				Nonfluent PWA (*N* = 63)			
	*M*	*SD*	Min	Max	*M*	*SD*	Min	Max
Age	59.94	12.23	24.42	83.76	59.06	14.16	26.34	91.92
Education (Years)	16.03	2.33	12	22	15.34	2.28	11	20
Post-Stroke Onset (Months)	68.50	63.84	4	295	51.94	44.48	3	187
WAB-R Fluency (out of 10)	7.48	1.44	6	9	4.13	1.09	1	5
WAB-R Auditory Comprehension (out of 10)	9.01	1.21	4.6	10	7.85	1.49	3.7	10
WAB-R Repetition (out of 10)	8.42	1.41	2.9	10	6.12	2.36	0	10
WAB-R Naming and Word Finding (out of 10)	8.45	1.54	0.9	10	6.37	2.21	0	9.1
WAB-R Aphasia Quotient (out of 100)	84.18	10.15	34.8	98	62.40	15.53	11.4	83

### 2.2 Task procedure

HC and PWA completed the same semantic fluency task either as a part of the Cognitive Linguistic Quick Test-Plus (CLQT+; Helm-Estabrooks, 2017) or the Western Aphasia Battery-Revised (WAB-R; Kertesz, 2006). During the semantic fluency task, participants were instructed to verbally produce as many animal category members as they could in 1 min. The semantic fluency task has been widely used to model group-based semantic networks (Siew et al., 2019).

### 2.3 Analyses

All data, analyses, and R Scripts for this project are available on the Open Science Framework: https://osf.io/4aufj/ (Kenett and Thompson-Schill, 2020).

#### 2.3.1 Semantic network estimation

We conducted two separate network analyses: (1) HC vs. PWA, and (2) fluent vs. nonfluent PWA. Semantic fluency data were analyzed using a semantic network approach (Borodkin et al., 2016; Kenett et al., 2013) where each node represents an animal category exemplar (e.g., *dog*) and edges represent semantic similarity between exemplars. All network analyses were conducted in R (version 4.4.1; R Core Team, 2024) using Christensen and Kenett's (2023) publicly available pipeline for semantic network analysis. More details regarding the individual steps of this approach are described below.

##### 2.3.1.1 Preprocessing

Semantic fluency responses were transcribed by the experimenter verbatim. The data were then cleaned and preprocessed using the *SemNetDictionaries* (version 0.2.0) and *SemNetCleaner* (version 1.3.4; Christensen and Kenett, 2023) packages. Non-category items (e.g., *book*) and perseverations were excluded, and variations of the same root word were standardized (e.g., *dogs* to *dog*). The clean data were then converted into a binary response matrix for network estimation using the *SemNetCleaner* package (version 1.3.4; Christensen and Kenett, 2023), where columns represent unique exemplars produced by the group and rows represent participants. A value of 1 indicates that a participant produced a given exemplar and a value of 0 indicates that the participant did not produce that specific exemplar. The finalized binary response matrix includes responses given by at least two participants in each group (HC vs. PWA; fluent vs. nonfluent PWA) to control for confounding influence of unique nodes and edges across group networks (Borodkin et al., 2016; Christensen et al., 2018; Cosgrove et al., 2021; Lebkuecher et al., 2024).

Given that PWA typically produce fewer responses than HC on average (e.g., Arroyo-Anlló et al., 2012; Basso et al., 1997; Bose et al., 2017, 2022; Faroqi-Shah and Milman, 2018), the binary response matrices were equated so that the networks for both group comparisons (HC vs. PWA; Fluent vs. Nonfluent PWA) were comprised of the same nodes. This step was necessary to prevent differences in the number of nodes from influencing network properties and biasing group comparisons (van Wijk et al., 2010). For HC and PWA network comparisons, this resulted in 97 nodes per network, with the exclusion of 117 idiosyncratic responses from HC and 23 from PWA. For fluent vs. nonfluent network comparisons, each network contained 46 nodes, with the elimination of 55 idiosyncratic responses from fluent PWA and 27 from nonfluent PWA.

##### 2.3.1.2 Network construction

Next, the *SemNet* package (version 1.4.4; Christensen and Kenett, 2023) was used to compute the semantic networks of semantic fluency responses, with edge weights between words in the network defined using the cosine similarity function. These networks are correlation-based because edge weights are estimated based on how often responses co-occur across the group. The cosine similarity function used in these analyses is related to Pearson’s correlation and is frequently used in analyses of textual corpora (Landauer and Dumais, 1997). Cosine similarity ranges from zero to one. Higher values indicate greater co-occurrence between two responses while a value of zero indicates that two responses never co-occur. Unlike Pearson’s correlation, cosine similarity values are always positive, eliminating the possibility of a negative associations between responses that do not co-occur.

The resulting word similarity matrix was then transformed into an *n x n* adjacency matrix of a weighted, undirected network. Each word in the matrix represents a node in the network and edges between two nodes represent the similarity between them. To refine the network and retain only the most meaningful connections, we applied the triangulated maximally filtered graph (Christensen et al., 2018; Massara et al., 2017) using the *NetworkToolbox* package (version 1.4.2; Christensen and Kenett, 2023). This method preserves strong correlations while reducing spurious connections, ensuring that the number of edges remains consistent across groups. Maintaining an equal number of nodes and edges across networks allows for direct group comparisons. Lastly, edge weightings were retained for subsequent percolation analyses.

##### 2.3.1.3 Network analysis

The *NetworkToolbox* package (version 1.4.2; Christensen and Kenett, 2023) was used to compute common network metrics for each group’s fluency data, specifically average shortest path length (ASPL; the average length of the shortest path between any pairs of nodes), clustering coefficient (CC; the likelihood that neighbors of a node will also be neighbors with each other), and modularity (Q; how well communities, or subgroups of nodes, are separable from each other in a network). Statistical comparisons were conducted using independent-samples *t*-tests to compare network metrics between groups (HC vs. PWA; fluent vs. nonfluent PWA).

In addition to global network measures, we also computed both local CC values (specifically Zhang and Horvath's (2005) weighted clustering coefficient) using the *clustZhang()* function and local degree for each node in the network (Opsahl et al., 2010) using the *centrality()* function in the *qgraph* package (version 1.9.8; Epskamp et al., 2012). Paired *t-*tests were used to assess differences between local characteristics of each group.

#### 2.3.2 Statistical analysis

##### 2.3.2.1 Random network comparison

To confirm that our estimated semantic networks exhibit meaningful, nonrandom structure, we statistically compared their parameters to those of simulated random networks with identical nodes and edges (Steyvers and Tenenbaum, 2005). Specifically, we generated 1,000 Erdös-Rényi random networks with a fixed edge probability (Erdös and Rényi, 1960) and calculated key network metrics (ASPL, CC, Q) to establish a reference distribution of random networks. Each estimated network was then assessed using a one-sample *z*-test against its respective reference distribution to evaluate statistical significance.

##### 2.3.2.2 Bootstrap analysis

Next, to enhance the robustness of group comparisons, a node-wise bootstrapping approach (Borodkin et al., 2016; Cosgrove et al., 2021; Kenett et al., 2016; Lebkuecher et al., 2024) was applied using the *SemNetCleaner* package (version 1.3.4; Christensen and Kenett, 2023) to simulate and compare partial semantic networks across participant groups. This method operates on the principle that if the observed differences between full networks are meaningful, they should persist across partial networks with the same nodes.

This procedure involves randomly selecting half of the nodes from the shared node set between groups, constructing distinct partial networks from these subsets of nodes, and computing key network metrics (ASPL, CC, Q). This process was repeated 1,000 times to simulate partial semantic networks, leading to a distribution of values for each network measure. Finally, independent-samples *t*-tests were conducted to compare network parameters between the bootstrapped partial networks and those estimated from the original dataset.

##### 2.3.2.3 Spreading activation simulation

We compared spreading activation between the HC and PWA networks and between the Fluent and Nonfluent PWA networks. Recall that the HC vs. PWA networks consisted of 97 nodes while the Fluent vs. Nonfluent PWA networks consisted of 46 nodes. We utilized the *spreadr* R package (version 0.2.0; Siew, 2019) to model how activation spreads in the networks. The following parameters were specified for the simulations. With this combination of parameters, we conducted simulations of spreading activation on our networks of interest (HC vs. PWA; Fluent vs. Nonfluent PWA).

An arbitrary *initial activation* value of 100 units was utilized for each word in our simulations (Lebkuecher et al., 2024; Vitevitch et al., 2011). *Decay (d)* refers to the proportion of activation lost at each time step. This parameter ranges from 0 to 1 and was set to 0 in the present simulations to align with parameter settings used in previous simulations (e.g., Lebkuecher et al., 2024; Siew, 2019; Vitevitch et al., 2011, 2023; Vitevitch and Mullin, 2021). *Retention (r)* refers to the proportion of activation that is retained in each node as it diffuses activation evenly to other nodes connected to it. This value ranges from 0 to 1 and was set to 0.5. The *suppress (s)* parameter in *spreadr* sets nodes with activation values lower than a selected value to activation = 0. Siew (2019) suggested this parameter be a very small value (e.g., <0.001). In the present simulations, suppress was set to 0 to be consistent with the parameter settings used in previous simulations (e.g., Lebkuecher et al., 2024; Siew, 2019; Vitevitch et al., 2011; Vitevitch and Mullin, 2021). *Time (t)* refers to the number of time steps that activation diffuses or spreads across the network. In the current simulations, time was set to 10 to remain consistent with previous work (e.g., Lebkuecher et al., 2024; Siew, 2019; Vitevitch et al., 2011). At the end of 10 time steps, we documented the final activation level of each word in the network.

To analyze the time course of activation, we examined activation levels across all 10 time steps. Paired *t*-tests were conducted to compare the final activation levels of words in the HC and PWA networks and the fluent and nonfluent PWA networks, focusing on node-level differences rather than overall group mean differences. Additionally, we explored the relationship between final activation levels of each seed node and local node characteristics (degree and clustering coefficient) using separate linear regression models for each group using the *lm*() function in R. In these models, final node activation was predicted by local degree and clustering coefficient, as these measures serve as proxies for semantic neighbors, which have been shown to impact word processing (e.g., Chen and Mirman, 2012; Mirman, 2011; Mirman and Magnuson, 2008).

##### 2.3.2.4 Percolation analysis

We also applied a percolation analysis to our data to examine the robustness and connectivity of the semantic networks by studying how the removal of nodes or edges affects the network structure. We applied a percolation algorithm to HC and PWA networks as well as the fluent and nonfluent PWA networks using the *CliquePercolation* package in R (version 0.4.0; Lange, 2021). The clique percolation method (Farkas et al., 2007; Palla et al., 2005) identifies overlapping communities, or subgraphs, of an entire weighted network. In the present analyses, we applied this method to the weighted semantic networks to define the number of k-cliques—fully connected clusters of k nodes. K-cliques that share at least two overlapping nodes are a community of k nodes.

To estimate the optimal number of k-clique communities for the semantic networks of healthy controls and patients with aphasia, as well as for fluent and nonfluent PWA, we used the *estimateNetwork()* function from the *bootnet* R package (version 1.6; Epskamp et al., 2018). We allowed for the smallest possible cliques (*k* = 3) to maximize sensitivity to the smallest possible communities. K-cliques were eliminated if the weighted connections between words fell below a predefined *intensity threshold* (*I*; Farkas et al., 2007), which ranged from 0.01 to 1, capturing minimum and maximum values for semantic relatedness. This range allows all nodes to be included at minimum values and all nodes to be excluded at the maximum value, thus capturing how nodes are retained across the range of semantic relatedness values. By incrementally increasing over iterations, we progressively removed overlapping connections between communities of semantically related words if they fell below the threshold until the networks could not break apart any further.

To quantify network cohesion, we computed the *area under the curve (AUC)* for the number of connected nodes across all *I* values. From this, we computed the percolation integral, which measures how quickly components within the network separate from the “giant component,” which refers to the largest cluster of interconnected nodes in the network (Kenett et al., 2018). A lower percolation integral indicates a steeper percolation slope, meaning the network is more susceptible to breaking apart. We ran 500 iterations of the clique percolation analysis to compare network cohesion between aphasia and control groups, as well as between fluent and nonfluent PWA. In each iteration, we computed the percolation integral for each group and conducted independent-samples *t*-tests to determine whether the mean percolation integrals significantly differed between groups.

## 3 Results

All analyses below focus on group comparisons between healthy controls (HC) and persons with aphasia (PWA) and between fluent PWA and nonfluent PWA. Additional three-group comparisons (HC vs. fluent PWA vs. nonfluent PWA) are reported in the Supplementary materials.

### 3.1 Semantic fluency performance

**Figure 1:**
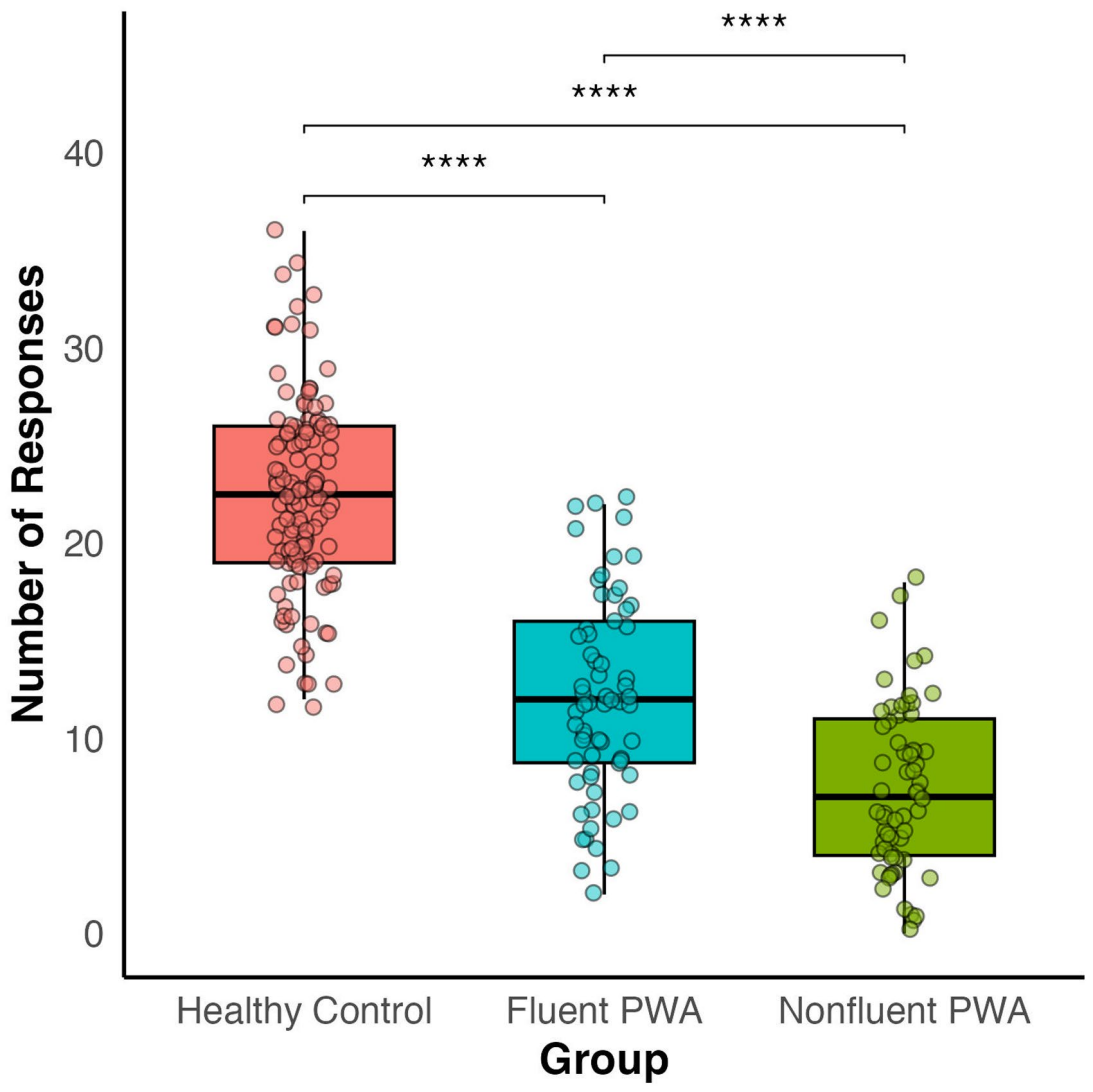
Group differences in number of appropriate semantic fluency responses.

We utilized simple linear regressions to examine whether HC and PWA differed in performance on the semantic fluency task by examining the total number of appropriate responses. To account for group differences in age and education, we included them as covariates in the model to verify that any group differences that emerged were not driven by demographic variability. As expected, we found that HC produced significantly more responses on the semantic fluency task (*M* = 22.47, *SD* = 4.90) relative to PWA (*M* = 9.63, *SD* = 5.25), and these group differences were significant even after controlling for age and education [*β* = −12.87, *t*(236) = −4.53, *p* < 0.001]. Additionally, fluent PWA produced significantly more responses (*M* = 11.95, *SD* = 5.08) than nonfluent PWA (*M* = 7.27, *SD* = 4.30), even after controlling for age and education [β = −4.06, *t*(117) = −5.08, *p* < 0.001; Figure 1].

### 3.2 Semantic network analyses

**Figure 2:**
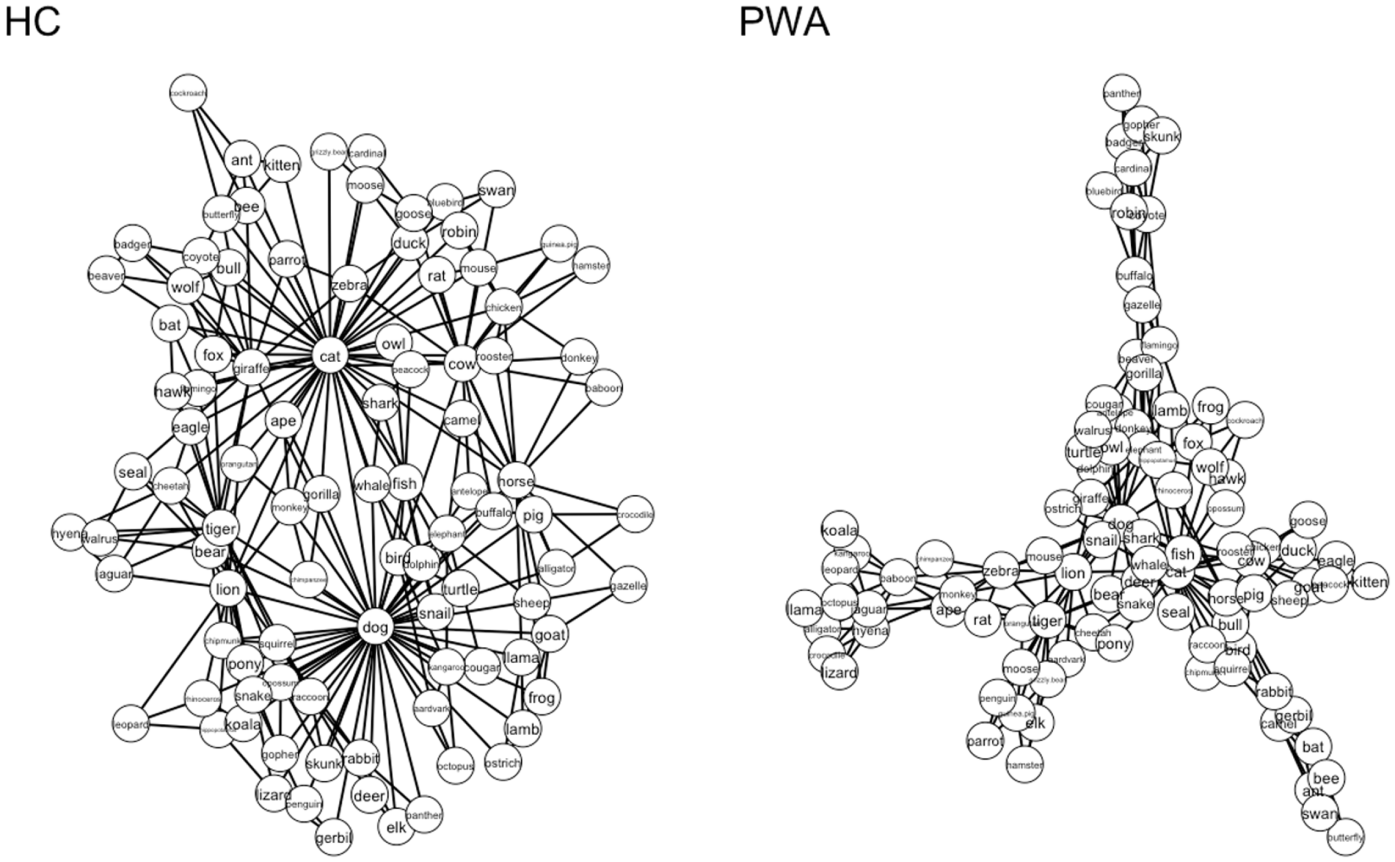
Two-dimensional visualization of HC and PWA semantic networks Based on verbal (semantic) fluency task. The estimated network for healthy controls (HC) is displayed on the left and the network estimated for persons with aphasia (PWA) is on the right. Each circle represents a word (node), and lines represent connections between words. Networks were all matched for number of nodes (97 nodes).

**Figure 3:**
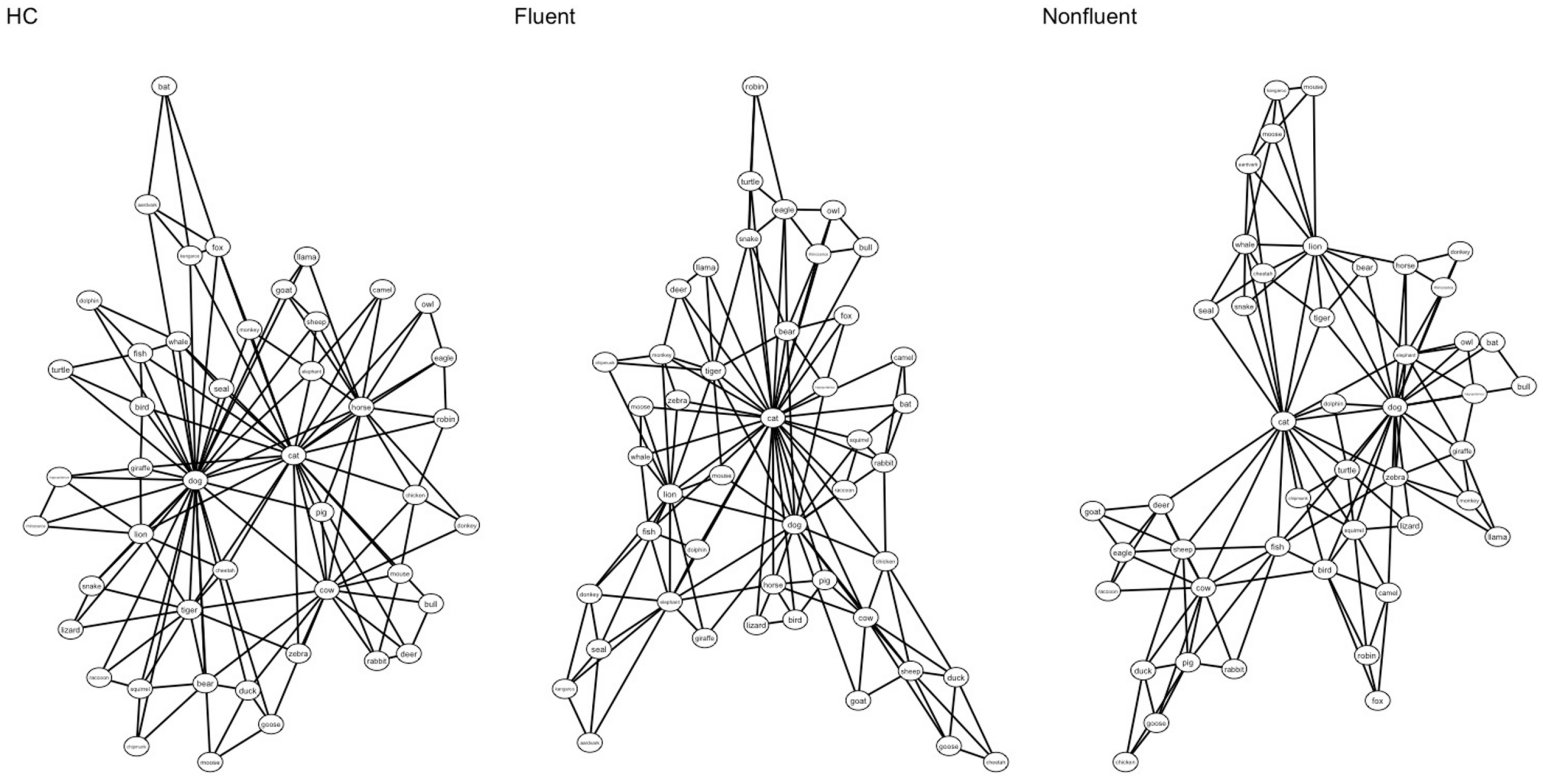
Two-dimensional visualization of HC, fluent PWA, nonfluent PWA semantic networks based on verbal (semantic) fluency task. The estimated network for HC is displayed on the left, fluent PWA in the middle, and nonfluent PWA on the right. Each circle represents a word (node), and lines represent connections between words. Networks were all matched for number of nodes (46 nodes).

Next, we estimated the semantic networks of the neurotypical controls versus PWA and fluent versus nonfluent PWA. The networks were visualized using the *compare.nets()* function from the *SemNet* package (version 1.4.4; Christensen and Kenett, 2023). In these 2D visualizations (Figures 2, 3), nodes are represented by circles and edges between them are represented by lines. These networks are undirected and weighted, meaning that the edges convey symmetrical (i.e., bidirectional) similarities between two nodes.

Visual comparison (Figure 2) suggests structural differences between the semantic networks of HC compared to PWA. To statistically validate our results, we first compared our modeled semantic networks to randomly generated graphs. The simulated random network analysis revealed that the graph theory metrics (ASPL, CC, and Q) for the HC and PWA groups were statistically different from random networks (all *p*’s < 0.001), with the exception of the comparison between the HC and random networks for Q (*p* = 0.96) and PWA and random network for ASPL (*p* = 0.06).

We then assessed differences in network metrics (ASPL, CC, and Q) across participant groups (Table 3). The HC semantic network was more interconnected than the PWA network, as evidenced by shorter ASPL [*t*(1998) = −50.58, *p* < 0.001, *d* = 2.26], higher CC [*t*(1998) = 54.29, *p* < 0.001, *d* = 2.43], and lower Q [*t*(1998) = −59.84, *p* < 0.001, *d* = 2.68], likely reflecting more efficient lexical retrieval in HC than PWA.

**Table 3:** HC vs. PWA global network metric comparisons.

Parameter	*M_HC_ (SD)*	*M_PWA_ (SD)*	*t-*statistic	*p-*value	Cohen’s *d*
ASPL	2.16 (0.09)	2.48 (0.18)	−50.58	**< 0.001**	2.26
CC	0.77 (0.01)	0.73 (0.02)	54.29	**< 0.001**	2.43
Q	0.41 (0.03)	0.49 (0.03)	−59.84	**< 0.001**	2.68

Means (M) and standard deviations (SD) of each group are presented for all network parameters. *t*-statistics, *p*-values, and Cohen’s d values are presented. Bold formatting indicates *p* < 0.05. Cohen’s d effect sizes (Cohen, 1988): 0.2—small; 0.5—medium; 0.8—large. HC, healthy control; PWA, persons with aphasia; ASPL, average shortest path length; CC, clustering coefficient; Q = modularity.

We next explored whether semantic network structure differs between different clinical aphasia profiles by comparing fluent vs. nonfluent PWA. Visual comparison (Figure 3) suggests structural differences between the semantic networks of fluent PWA compared to nonfluent PWA. To statistically validate our results, we compared our modeled semantic networks to randomly generated graphs. The simulated random network analysis revealed that the graph theory metrics (ASPL, CC, and Q) for the fluent and nonfluent PWA groups were statistically different from random networks (all *p*’s < 0.05), with the exception of the comparison between the fluent PWA network and random networks for Q (*p* = 0.43).

We then assessed differences in network metrics (ASPL, CC, and Q) across participant groups (Table 4). The fluent PWA semantic network, compared to the nonfluent PWA semantic network, was characterized by shorter ASPL [*t*(1998) = 20.71, *p* < 0.001, *d* = 0.93], higher CC [*t*(1998) = 20.71, *p* < 0.001, *d* = 0.93], and lower Q [*t*(1998) = 20.71, *p* < 0.001, *d* = 0.93].

**Table 4:** Fluent PWA vs. nonfluent PWA global network metric comparisons.

Parameter	*M_Fluent_ (SD)*	*M_Nonfluent_ (SD)*	*t-*statistic	*p-*value	Cohen’s *d*
ASPL	1.92 (0.08)	2.00 (0.09)	−20.71	**< 0.001**	0.93
CC	0.75 (0.01)	0.74 (0.03)	15.72	**< 0.001**	0.70
Q	0.32 (0.03)	0.34 (0.03)	−12.68	**< 0.001**	0.57

Means (M) and standard deviations (SD) of each group are presented for all network parameters. *t*-statistics, *p*-values, and Cohen’s d values are presented. Bold formatting indicates *p* < 0.05. Cohen’s d effect sizes (Cohen, 1988): 0.2—small; 0.5—medium; 0.8—large. ASPL, average shortest path length; CC, clustering coefficient; Q, modularity.

Altogether, compared to the nonfluent PWA group, the lexical-semantic network of the fluent PWA group was significantly more connected (higher CC), with shorter average paths (shorter ASPL), and fewer communities (lower Q). Thus, the fluent PWA network was on average more interconnected than the nonfluent PWA network, with PWA networks being less interconnected than the HC network.

Thus far we have only focused on global network measures that describe the overall structure and organization of the entire network. However, we also compared local node measures that describe properties of individual nodes (words) and their immediate connections. We examined local degree (the number of connections a word has to other words in the network) and local clustering coefficient (the likelihood that neighbors of a node will also be neighbors with each other) values for individual words in each network. Significant differences emerged between HC and PWA networks with the PWA network exhibiting lower degree [*t*(96) = 2.14, *p* = 0.04, *d* = 0.23] and lower local clustering coefficient (*t*(96) = 5.81, *p* < 0.0001, *d* = 0.59) than the HC network. Comparing between fluent and nonfluent PWA networks, we also observed differences in local clustering coefficient, with lower clustering coefficient for the nonfluent PWA network compared to the fluent PWA network [*t*(45) = 3.37 *p* = 0.002, *d* = 0.50], mirroring what we observed in our analysis of global CC. In contrast, there was no significant difference in degree between the nonfluent and fluent PWA networks [*t*(45) = 1.16, *p* = 0.25, *d* = 0.17; Tables 5, 6].

**Table 5:** HC vs. PWA local network metric comparisons.

Parameter	*M_HC_ (SD)*	*M_PWA_ (SD)*	*t-*statistic	*p-*value	Cohen’s *d*
Degree	2.36 (3.12)	1.93 (1.61)	2.14	**0.04**	0.23
Clustering coefficient	0.37 (0.13)	0.27 (0.11)	5.81	**< 0.001**	0.59

Means (M) and standard deviations (SD) of each group are presented for all network parameters. *t*-statistics, *p*-values, and Cohen’s d values are presented. Bold formatting indicates *p* < 0.05. Cohen’s d effect sizes (Cohen, 1988): 0.2—small; 0.5—medium; 0.8—large.

**Table 6:** Fluent PWA vs. nonfluent PWA local network metric comparisons.

Parameter	*M_Fluent_ (SD)*	*M_Nonfluent_ (SD)*	*t-*statistic	*p-*value	Cohen’s *d*
Degree	2.30 (2.28)	2.06 (1.53)	1.16	0.25	0.17
Clustering coefficient	0.34 (0.13)	0.28 (0.10)	3.37	**< 0.01**	0.50

Means (M) and standard deviations (SD) of each group are presented for all network parameters. *t*-statistics, *p*-values, and Cohen’s d values are presented. Bold formatting indicates *p* < 0.05. Cohen’s d effect sizes (Cohen, 1988): 0.2—small; 0.5—medium; 0.8—large.

### 3.3 Spreading activation simulation

**Figure 4:**
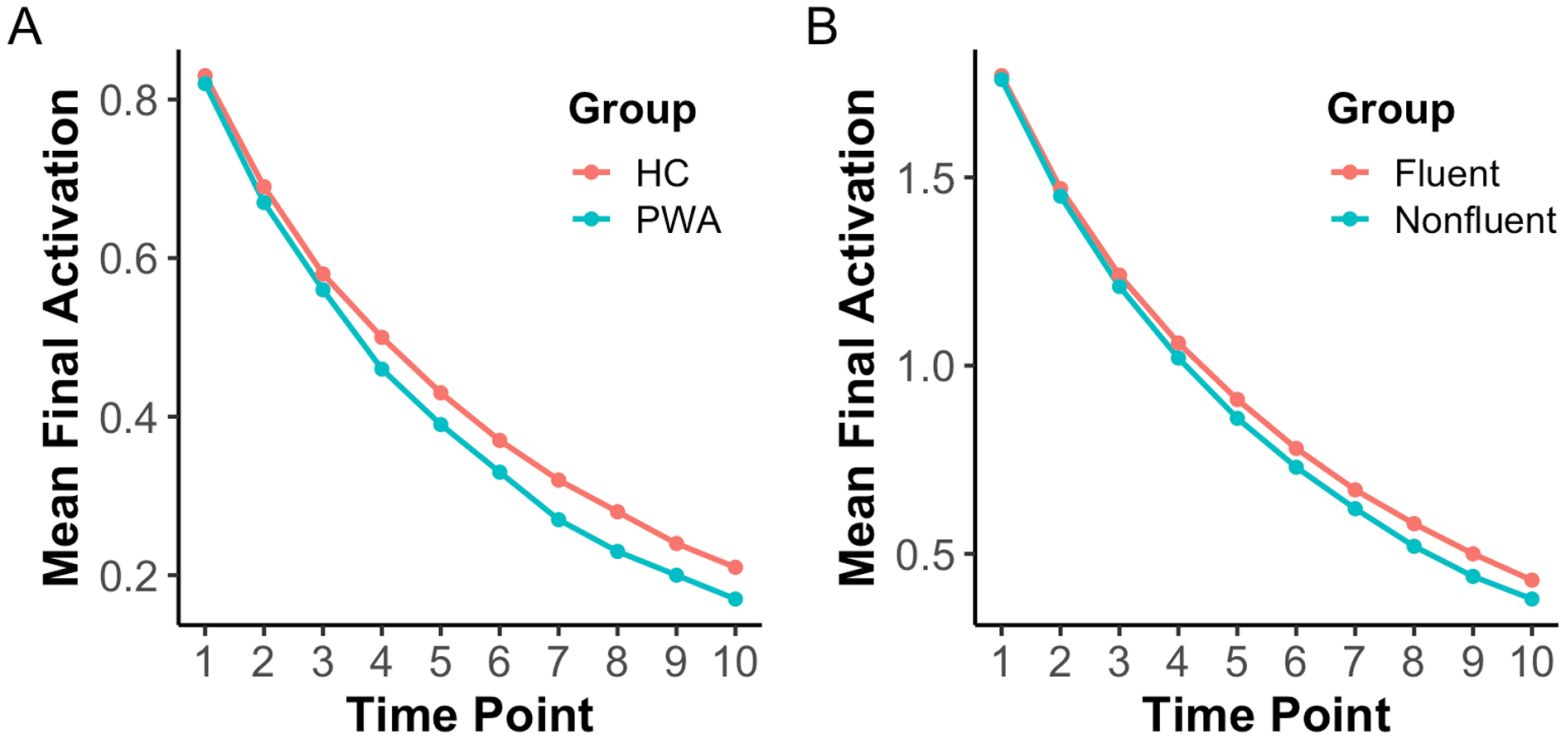
HC vs. PWA networks and fluent vs. nonfluent PWA comparisons of final activation from spreading activation simulation.

To further delineate the relationship between different network metrics and processing efficiency, we implemented a spreading activation simulation on each network as well. Paired *t*-tests conducted across 10 time points revealed that individuals with aphasia exhibited lower final activation levels than healthy controls at time points 4–10 (*p*’s < 0.01; see Table 7; Figure 4A). A similar pattern of results is observed when implementing a spreading activation simulation on the fluent and nonfluent PWA networks. Nonfluent PWA exhibit lower final activation levels than fluent PWA at time points 6–10 (*p*’s < 0.05; see Table 8; Figure 4B). These findings indicate that spreading activation is weaker in individuals with aphasia compared to neurotypical adults, with nonfluent PWA exhibiting weaker spreading activation than their fluent counterparts.

**Table 7:** HC vs. PWA comparisons of final activation from spreading activation simulation.

Time Point	*M_HC_ (SD)*	*M_PWA_ (SD)*	*t*-value	*p*-value
1	0.83 (5.22)	0.82 (5.20)	0.36	0.72
2	0.69 (3.08)	0.67 (3.05)	0.88	0.38
3	0.58 (2.02)	0.56 (2.00)	1.62	0.11
4	0.50 (1.43)	0.46 (1.42)	2.58	**<0.01**
5	0.43 (1.06)	0.39 (1.06)	3.79	**<0.001**
6	0.37 (0.81)	0.33 (0.82)	5.21	**<0.0001**
7	0.32 (0.64)	0.27 (0.64)	6.85	**<0.0001**
8	0.28 (0.51)	0.23 (0.51)	8.68	**<0.0001**
9	0.24 (0.42)	0.20 (0.41)	10.67	**<0.0001**
10	0.21 (0.35)	0.17 (0.33)	12.81	**<0.0001**

Bold formatting indicates *p* < 0.05.

**Table 8:** Fluent vs. nonfluent PWA comparisons of final activation from spreading activation simulation.

Time Point	*M_Fluent_ (SD)*	*M_Nonfluent_ (SD)*	*t*-value	*p*-value
1	1.77 (7.47)	1.76 (7.47)	0.06	0.95
2	1.47 (4.38)	1.45 (4.41)	0.26	0.80
3	1.24 (2.87)	1.21 (2.91)	0.55	0.58
4	1.06 (2.03)	1.02 (2.06)	0.95	0.34
5	0.91 (1.51)	0.86 (1.53)	1.46	0.14
6	0.78 (1.16)	0.73 (1.17)	2.09	**0.04**
7	0.67 (0.92)	0.62 (0.91)	2.84	**0.005**
8	0.58 (0.75)	0.52 (0.72)	3.69	**< 0.0001**
9	0.50 (0.61)	0.44 (0.57)	4.63	**< 0.0001**
10	0.43 (0.51)	0.38 (0.46)	5.65	**< 0.0001**

Bold formatting indicates *p* < 0.05.

Given that semantic neighbors have been shown to impact word processing (e.g., Chen and Mirman, 2012; Mirman, 2011; Mirman and Magnuson, 2008), we investigated whether node degree and clustering coefficient significantly predicted final activation. For both HC and PWA networks, local degree and local clustering coefficient were significant predictors of the final activation values, such that words with high degrees and lower clustering coefficients had higher final activations (*p*’s < 0.001; see Table 9). These results suggest that although the simulated activation spread more slowly among PWA, the effects of degree and clustering coefficient on simulated activation were similar for PWA and HC.

**Table 9:** Summary of linear regression models predicting each group network’s final activation based on node degree and clustering coefficient.

Parameter	Estimate	SE	*t*-value	*p*-value
Model 1: HC				
Intercept	0.92	0.02		
Degree	0.43	0.03	16.78	**< 0.001**
Clustering Coefficient	−0.17	0.03	−6.43	**< 0.001**
Model 2: PWA				
Intercept	1.10	0.04		
Degree	0.35	0.04	7.88	**< 0.001**
Clustering Coefficient	−0.16	0.04	−3.57	**< 0.001**
Model 3: Fluent PWA				
Intercept	1.14	0.03		
Degree	0.55	0.03	17.25	**< 0.001**
Clustering Coefficient	−0.16	0.03	−5.02	**< 0.001**
Model 4: Nonfluent PWA				
Intercept	1.26	0.04		
Degree	0.44	0.05	9.27	**< 0.001**
Clustering Coefficient	−0.09	0.05	−1.78	0.08

Bold formatting indicates *p* < 0.05.

When comparing fluent and nonfluent PWA networks, node degree was the only significant predictor of final activation values in both groups (*p*’s < 0.001), with high degree words exhibiting greater final activation across both fluent and nonfluent PWA networks. However, differences emerged in the effect of local clustering coefficient. While clustering coefficient significantly predicted final activation in the fluent PWA network (*p* < 0.001), it did not reach significance in the nonfluent PWA network (*p* = 0.08; see Table 9) but is in the same direction as the fluent PWA group.

### 3.4 Percolation analyses

**Figure 5:**
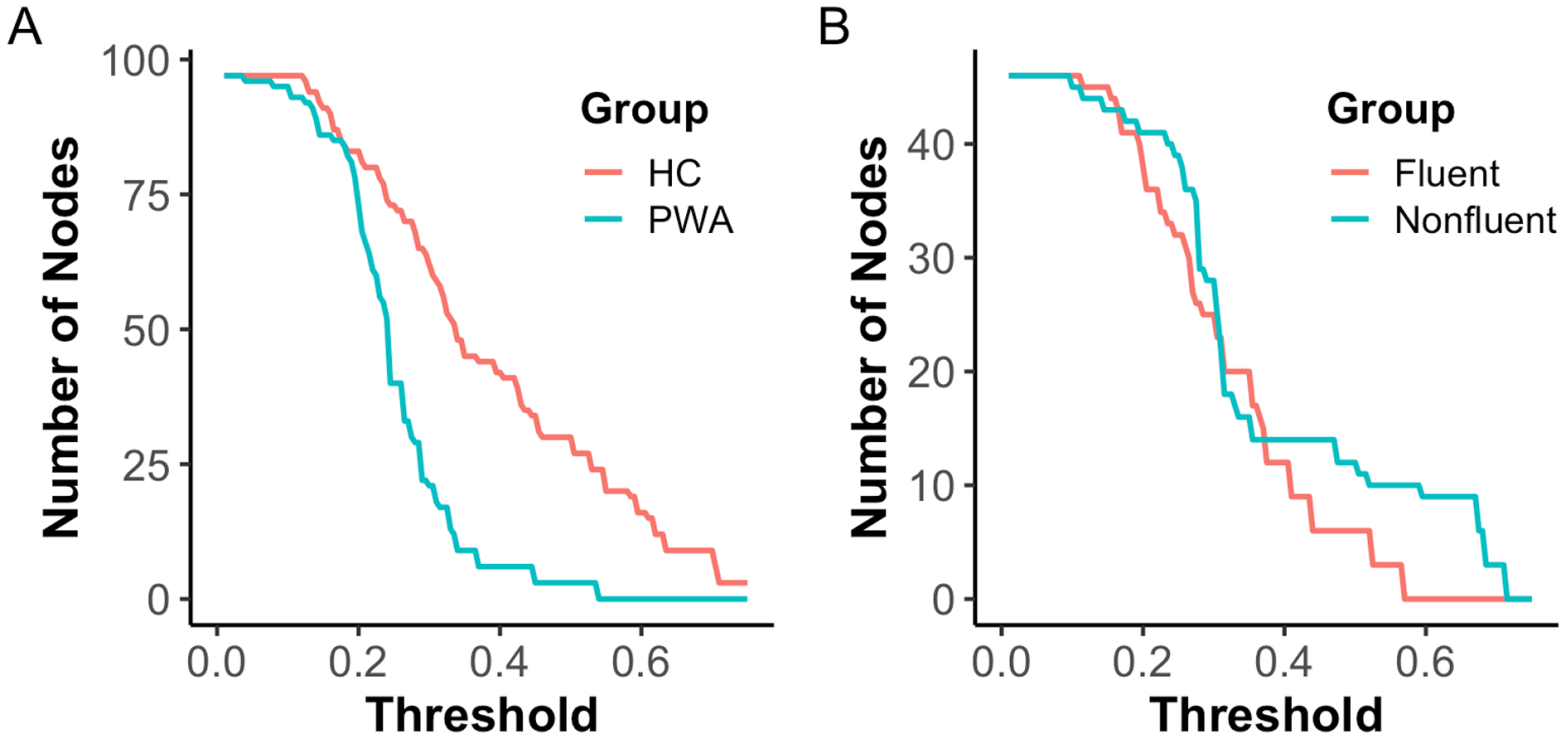
Percolation analysis of HC vs. PWA networks and fluent vs. nonfluent PWA networks. The total number of nodes differs in each group comparisons. HC and PWA networks contain 97 nodes while fluent and nonfluent PWA networks contain 46 nodes.

To assess network resilience, we performed a percolation analysis, which measures how quickly a network breaks apart under progressive node removal. A higher percolation integral indicates a slower breakdown, reflecting a more robust network structure. Results revealed that the HC network was significantly more resilient (*M* = 49.99, *SD* = 1.69) compared to the PWA network (*M* = 43.12, *SD* = 1.13; *t*(998) = 75.54, *p* < 0.001). When comparing fluency subgroups, the fluent PWA network showed greater resilience (*M* = 23.88, *SD* = 1.04) than the nonfluent PWA network (*M* = 21.83, *SD* = 0.66; *t*(998) = 37.15, *p* < 0.001). Figure 5 illustrates an example of the number of connected nodes in each group network at increasing intensity with a steeper slope indicating a lower percolation integral and less robust network structure. Nonfluent PWA exhibited the least stable networks, fragmenting more rapidly than their fluent counterparts (Figure 5B), while PWA demonstrated weaker network integrity than HC (Figure 5A).

## 4 Discussion

The current study applied semantic network analysis to verbal (semantic) fluency data from a large cohort of individuals with post-stroke aphasia and healthy controls, providing the first large-scale examination of semantic network structure in aphasia. By constructing group-level lexical-semantic networks for controls, fluent PWA, and nonfluent PWA, we examined how network structure differs as a function of clinical status and aphasia subtype. We quantified global network properties (average shortest path length, clustering coefficient, and modularity), simulated spreading activation to model lexical access efficiency, and conducted percolation analysis to assess network resilience.

### 4.1 Lexical-semantic network disruptions in aphasia

Our findings provide compelling evidence that the lexical-semantic network is systematically disrupted in post-stroke aphasia. As predicted, the lexical-semantic network of PWA exhibited longer average shortest path length (ASPL), lower clustering coefficients (CC), and higher modularity (Q) compared to the lexical-semantic network of healthy controls. These features indicate that the network is less efficiently structured because of less cohesion and more fragmentation for PWA. These disruptions were most pronounced in nonfluent PWA, whose networks were significantly less integrated than those of fluent PWA, suggesting a gradation of impairment consistent with fluency-based clinical profiles. To better understand how these networks function dynamically, we simulated spreading activation and conducted percolation analyses. Spreading activation simulations revealed that activation spread more slowly and to fewer nodes in PWA networks, particularly in the nonfluent PWA network, suggesting reduced efficiency in lexical access. Percolation analyses, which test the flexibility of a network by removing nodes based on their connectivity, showed that PWA networks were more vulnerable to fragmentation, indicating less resilient network architecture.

To examine how local network structure shapes lexical activation, we also analyzed whether node-level metrics like degree and local clustering coefficient predicted final activation values in our simulations. Consistent with prior work demonstrating the role of semantic neighbors in lexical access (e.g., Chen and Mirman, 2012; Mirman, 2011; Mirman and Magnuson, 2008), both degree and clustering coefficient significantly predicted final activation in the healthy control and PWA networks. Words with more connections (higher degree) and less tightly clustered neighbors (lower clustering coefficient) received higher activation, supporting the view that connectivity and local structure facilitate lexical access. Interestingly, this relationship differed between fluent and nonfluent PWA. While degree remained a significant predictor of activation in both groups, clustering coefficient prediction activation only for fluent PWA. This divergence may reflect differences in the local organization or efficiency of activation spread in more severely impaired lexical systems, underscoring the value of simulation-based approaches in capturing nuanced subgroup-specific disruptions.

These results are consistent with interactive models of lexical retrieval (Dell et al., 1997; Martin and Dell, 2019), which posits that successful word retrieval depends not only on the integrity of the lexical-semantic network but also on the efficiency of activation spread through that network. Our findings suggest that lexical retrieval impairments in aphasia are linked to both weakened spreading activation and disruptions in the structural architecture of the mental lexicon. Unlike traditional verbal fluency metrics (e.g., total number of responses, clustering and switching behaviors), semantic network analyses reveal that lexical retrieval impairments in aphasia reflect both inefficient activation dynamics and fragility in network structural, a dual disruption not readily captured by traditional verbal fluency metrics. It is important to note that these findings do not imply that lexical representations are absent in aphasia (Mirman and Britt, 2014; Rapp and Caramazza, 1993; Warrington and Shallice, 1984). Rather, because all networks were constructed using the same set of words (nodes) across groups, the key difference lies in how these words are connected. That is, the underlying network structure may constrain the ability to access and retrieve words efficiently.

Notably, our results revealed reliable differences between fluent and nonfluent PWA, differences that have not been consistently observed in previous clustering and switching analyses. While an earlier case study suggested that nonfluent PWA exhibit more intact clustering but reduced switching (Baldo et al., 2010), larger group studies have failed to find differences in clustering and switching behaviors between fluent and nonfluent subtypes (Bose et al., 2017). In contrast, we observed clear differences across multiple structural metrics: the nonfluent PWA network was less integrated and more fragmented than the fluent PWA network. Additionally, these distinctions were mirrored in our spreading activation simulations and percolation analyses. Our findings suggest semantic network analysis may offer greater sensitivity to detecting clinically meaningful subgroup differences, potentially providing a more sensitive index of lexical-semantic impairment.

### 4.2 Limitations and future directions

The current approach relied on comparisons of group-level semantic networks, meaning that networks were constructed by aggregating responses across participants within each group. This enabled us to compare lexical-semantic organization across broad clinical contrasts, namely healthy controls vs. PWA and fluent vs. nonfluent PWA. In network analysis, it is generally expected that observed semantic networks will differ from random networks, reflecting meaningful underlying structure. We note that the average shortest path length for the PWA network and modularity for the control network did not statistically differ from random networks and should be interpreted with caution. These null findings may reflect reduced efficiency in PWA and less distinctive modular structure in controls, but they could also stem from methodological factors inherent to group-level network construction. Group-level aggregation obscures individual variability which can result in denser, more homogeneous networks (Zemla and Austerweil, 2018). Relatedly, the sparsity of fluency data for PWA could have also impacted the emergence of a strong community structure. Together, characteristics of our dataset and methodological constraints may have led to reduced differences between control and PWA networks versus their degree-matched random networks.

We also note that healthy controls and PWA were not matched on sex, age, or education, but this demographic variability did not drive the primary behavioral differences observed. After controlling for age and education, group differences in the total number of appropriate responses remained significant, suggesting that demographic variability is not driving group differences, at least not in overall semantic fluency performance. Nevertheless, the two groups differed in significantly in various demographic factors: the PWA group included a greater proportion of men, and men have been reported to produce more animals than women during animal fluency tasks (Hirnstein et al., 2023; but see also Lanting et al., 2009; Scheuringer et al., 2017). The PWA group was also significantly younger compared to the controls, which could confer an advantage given younger adults typically produce a greater number of appropriate items and exhibit more interconnected and efficient semantic network structure (Cosgrove et al., 2021, 2023; Wulff et al., 2022b). In contrast, the PWA group reported fewer years of education than controls, which has been associated with poorer verbal fluency performance (Beran et al., 2024). Altogether, these demographic factors may exert opposing influences on semantic fluency performance and network structure. The extent to which age, education, and other demographic factors influence network structure remains an open question, and future work including individual-level networks will be important for clarifying how these variables contribute to variability in network organization.

Relatedly, prior research has shown that differences observed in aggregate networks are not always preserved in individual networks, particularly in populations with high heterogeneity (Cosgrove et al., 2023; Jeong and Hills, 2024; Wulff et al., 2022b). Given the heterogeneity of aphasia, focusing on individual-level networks would allow for finer-grained assessment of how demographic and structural variables (e.g., network size) impact network organization and will be critical for clinical assessment and for tracking how an individual’s semantic network might change over time in response to treatment. Additionally, group-level semantic networks do not account for temporal sequence of individual responses, and we acknowledge that capturing these temporal dynamics would further inform out understanding of lexical retrieval processes (Bertola et al., 2014; Zemla and Austerweil, 2018, 2019). Taken together, the limitations of the current study highlight the importance of examining individual-level networks, which are better suited to capture individual variability. Despite this recognition, there is a need for developing methods to capture individual semantic networks (Wulff et al., 2022a), especially for clinical populations. There are ongoing efforts to construct individual networks from other tasks such as relatedness judgments (Benedek et al., 2017; Cosgrove et al., 2023; He et al., 2020; Ovando-Tellez et al., 2022) and free associations (Kenett et al., 2014; Nelson et al., 2004), albeit in primarily healthy populations.

It is worth noting that semantic fluency tasks traditionally rely on concrete, highly imageable categories (Castro et al., 2021), with the animal category being the most common. The current study focused on animals given its prevalence in standardized clinical and language assessments. However, the generalizability of a semantic network approach would be strengthened by expanding to other lexical categories (and beyond concrete nouns more broadly). While recent work has expanded this space to include more abstract semantic categories (Cosgrove et al., 2026), another area of growing interest that expands beyond noun production is action fluency (i.e., verbs). Although action fluency has been investigated using traditional scoring methods in other clinical populations, such as Parkinson’s disease and Alzheimer’s disease (see Beber and Chaves, 2014; Gianelli et al., 2021 for reviews), it remains largely understudied in aphasia (but see Faroqi-Shah and Milman, 2018; Scheffel et al., 2021). Verb access is crucial for sentence production and grammatical encoding, and constructing semantic networks from action fluency responses could offer unique insight into the organization of verb representation in aphasia. Qiu et al. (2021) compared noun and verb networks in healthy adults and found that verb networks were more condensed and less modular than noun networks, suggesting less structured organization for verbs. Consequently, damage to verb network structure could lead to disproportionate difficulties with verb retrieval since disruptions to a densely interconnected system could impair efficient access and increase competition among related verbs. This may explain why some individuals with aphasia show poorer performance on action fluency compared to noun fluency (Faroqi-Shah and Milman, 2018; Scheffel et al., 2021).

Another point of future consideration is that in the current study aphasia severity and aphasia type (fluent vs. nonfluent) are closely related, and it is difficult to discern whether the group-level differences in network structure truly reflect differences between fluent and nonfluent PWA or whether they were driven by severity. As such, extending network-based approaches to syntax represents an important avenue for future research. Syntactic networks, which capture how words are combined into larger structural units, may be especially sensitive to clinical characteristics not fully reflected in lexical-semantic networks. Importantly, syntactic networks afford us the opportunity to incorporate disfluencies as nodes within the network, providing a richer representation of how breakdowns in fluency may interact with language processes. Examining syntactic networks could provide insights into discourse-level impairments in aphasia and help identify treatment targets that address not only lexical retrieval but also the organization of sentence and discourse production.

## 5 Conclusion

The present study applied a network science approach to semantic fluency data to examine how lexical-semantic organization differs between healthy controls and persons with aphasia, including fluent and nonfluent aphasia subtypes. Our analyses of group-level semantic networks revealed that PWA exhibited longer path lengths, lower clustering, and higher modularity, which are indicative of less efficient, less cohesive, and less flexible network structures. These disruptions were most pronounced in nonfluent PWA. Spreading activation simulations and percolation analyses further revealed the PWA networks were less efficient and less structurally robust. Together, these findings suggest lexical retrieval impairments may affect both degraded network structure and impaired activation dynamics. More broadly, this work underscores the value of network science as a powerful framework for quantifying lexical organization and identifying meaningful variation across clinical profiles. Changes in network structure can also be leveraged to examine the efficacy of treatment or intervention. Indeed, a growing body of research demonstrates that semantic network structure is sensitive to experience-dependent change, with interventions ranging from creativity training to problem-solving tasks inducing measurable shifts in network typology (Bieth et al., 2024; Kenett and Thompson-Schill, 2020; Lai et al., 2024). These findings highlight the potential of network analysis not only for characterizing baseline network organization, but also for tracking how that organization evolves in response to targeted interventions in aphasia.

## Data Availability

The datasets presented in this study can be found in online repositories. The names of the repository/repositories and accession number(s) can be found in the article/Supplementary material.
