# Supplementary material for: Lexical retrieval in fluent and nonfluent aphasia: a network analysis of verbal fluency data
Source: Front Hum Neurosci. 2025 Dec 5;19:1710907. doi: 10.3389/fnhum.2025.1710907 (PMC12714882; doi:10.3389/fnhum.2025.1710907)

**Supplemental Materials**

**Table S1**

*HC vs Fluent PWA vs Nonfluent PWA Global Network Metric Comparisons*

| **Parameter** | ***M_HC_ (SD)*** | ***M_Fluent_ (SD)*** | ***M_Nonfluent_ (SD)*** | ***F*(2, 2997)** | ***p*-value** | ***Partial*** *η²* |
| --- | --- | --- | --- | --- | --- | --- |
| ASPL | 1.84 (0.05) | 1.92 (0.08) | 2.00 (0.09) | 1060.13 | **< .001** | .41 |
| CC | 0.76 (0.01) | 0.75 (0.01) | 0.73 (0.03) | 598.18 | **< .001** | .33 |
| Q | 0.29 (0.03) | 0.33 (0.04) | 0.34 (0.03) | 841.38 | **< .001** | .39 |

**Table S2**

*Pairwise Comparisons of Global Network Metrics*

| **Parameter** | **Comparison** | **Mean Difference** | **95% CI** | ***p_adj_*** |
| --- | --- | --- | --- | --- |
| ASPL | HC vs. Fluent | –0.080 | [–0.088, –0.072] | **< .001** |
|  | HC vs. Nonfluent | –0.159 | [–0.167, –0.151] | **< .001** |
|  | Fluent vs. Nonfluent | –0.079 | [­–0.087, –0.071] | **< .001** |
| CC | HC vs. Fluent | 0.013 | [0.011, 0.015] | **< .001** |
|  | HC vs. Nonfluent | 0.027 | [0.025, 0.028] | **< .001** |
|  | Fluent vs. Nonfluent | 0.014 | [0.012, 0.015] | **< .001** |
| Q | HC vs. Fluent | –0.039 | [–0.043, –0.036] | **< .001** |
|  | HC vs. Nonfluent | –0.055 | [–0.058, –0.051] | **< .001** |
|  | Fluent vs. Nonfluent | –0.016 | [–0.019, –0.012] | **< .001** |

**Figure S1**

*Percolation analysis of HC vs Fluent PWA vs Nonfluent PWA networks*


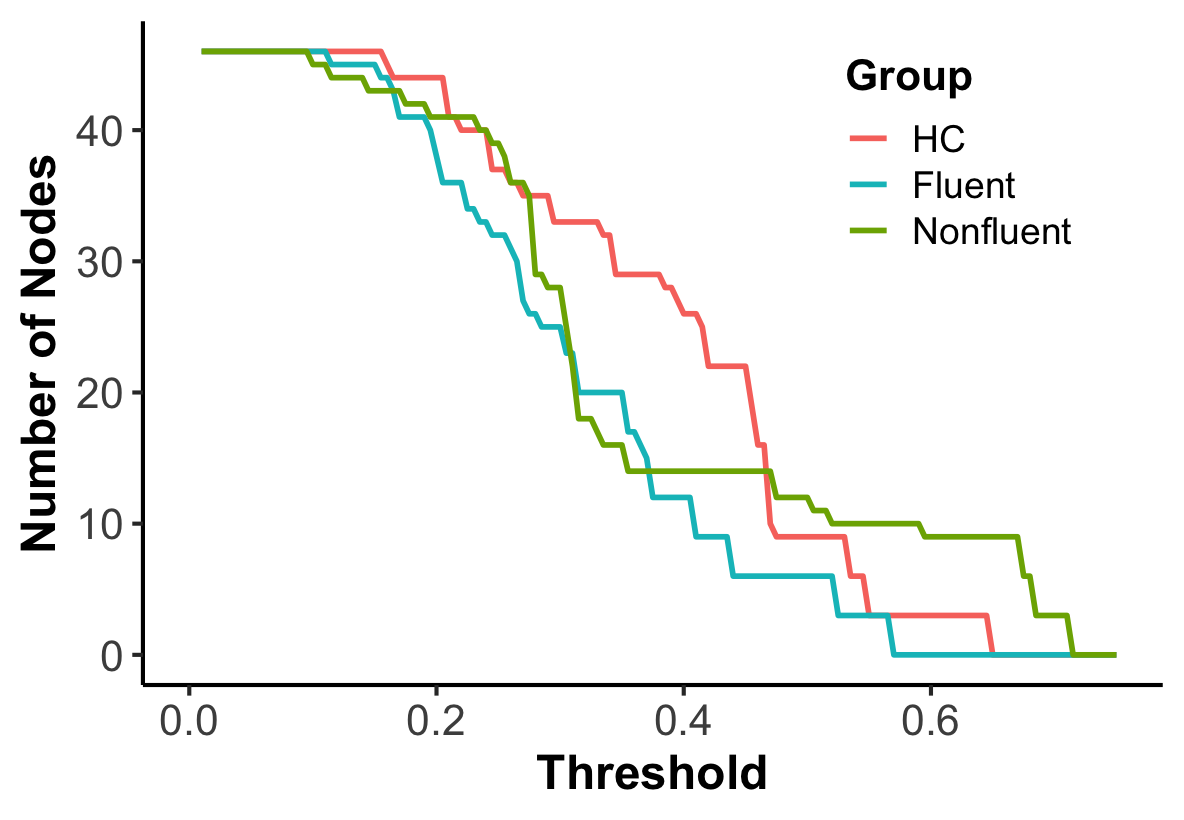


**Table S3**

*Pairwise Comparisons of Percolation Area Under the Curve*

| **Comparison** | **Mean Difference** | **95% CI** | ***t*-value** | ***p*-value** |
| --- | --- | --- | --- | --- |
| HC vs. Fluent | 3.99 | [1.31, 5.35] | 51.19 | **< .001** |
| HC vs. Nonfluent | 6.10 | [3.29, 7.27] | 87.15 | **< .001** |
| Fluent vs. Nonfluent | 2.10 | [­0.29, 3.22] | 36.36 | **< .001** |

**Figure S2**

*Spreading Activation Over Time Points Across Groups*


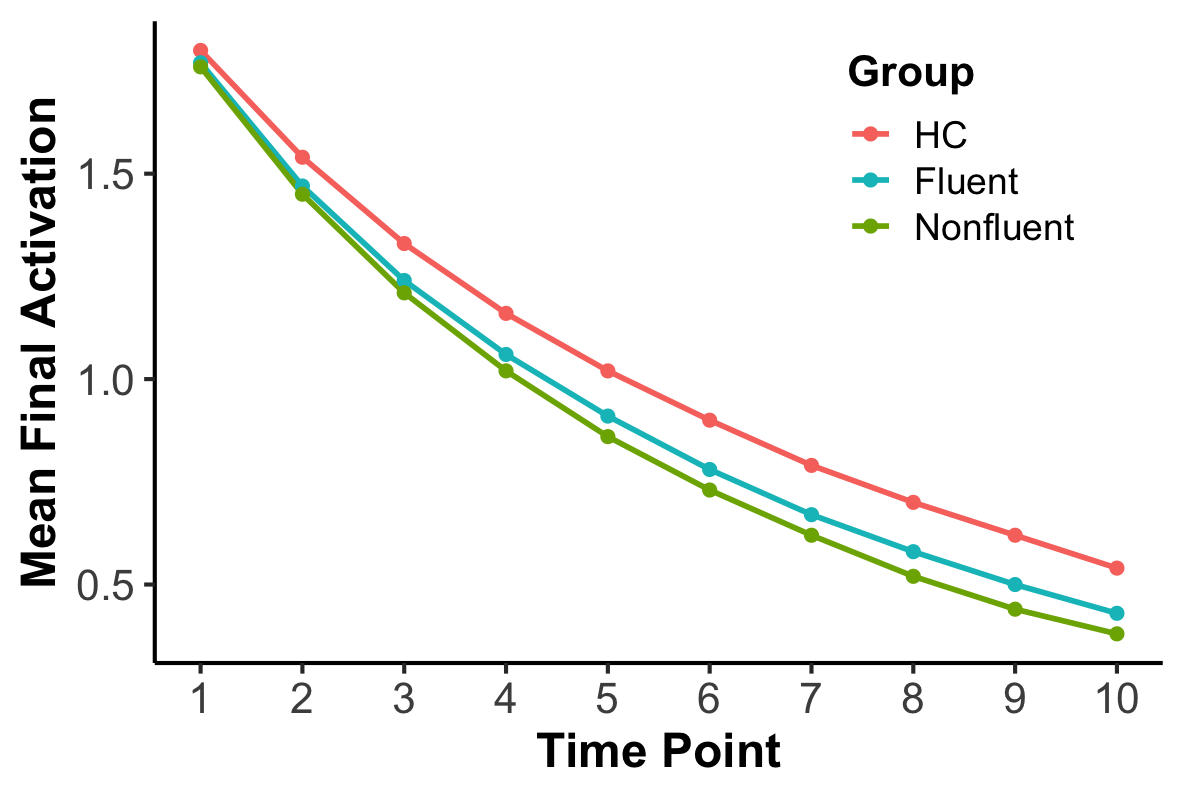

Supplement: Supplementary file 1 [file Supplementary_file_1.docx]
